# Reply to: “Re-evaluating the evidence for a universal genetic boundary among microbial species”

**DOI:** 10.1038/s41467-021-24129-1

**Published:** 2021-07-07

**Authors:** Luis M. Rodriguez-R, Chirag Jain, Roth E. Conrad, Srinivas Aluru, Konstantinos T. Konstantinidis

**Affiliations:** 1 School of Civil and Environmental Engineering, and School of Biological Sciences, Georgia Institute of Technology, Atlanta, GA USA; 2 Department of Microbiology, and Digital Science Center (DiSC), University of Innsbruck, Innsbruck, Tyrol Austria; 3 Department of Computational and Data Sciences, Indian Institute of Science, Bengaluru, India; 4 School of Computational Science and Engineering, and Institute for Data Engineering and Science, Georgia Institute of Technology, Atlanta, GA USA

**Keywords:** Classification and taxonomy, Applied microbiology

**Replying to** C. S. Murray et al. *Nature Communications* 10.1038/s41467-021-24128-2 (2021)

It has recently been reported^1^ that when one removes multiple genomes within a named species or species defined at the 95% genome-aggregate average nucleotide identity (ANI) level by sampling one genome per species or only two genomes that maximize the represented diversity within the species^1^, the ANI discontinuity (or “ANI gap”) observed between species based on all available genomes is lost. [Discontinuity or gap here refers to the small number of genome pairs showing 85–95% ANI relative to counts of pairs showing >95% and <85% ANI]. In other words, the bimodal distribution of ANI values previously observed by comparing the ~90,000 genomes available in the NCBI database^2^ disappears. These results also echo earlier findings by others based on different genome datasets [e.g^3^,]. Murray and colleagues have interpreted these results as evidence that a natural genetic discontinuity (i.e., an ANI boundary) between species may not exist, and the previously observed ANI boundary could simply be the result of isolation biases that favor redundant (or closely related) organisms. Note that there is no universally accepted species concept for prokaryotes (i.e., what a species is) but there are widely used standards on how to name species (i.e., a working species definition)^4^. The ~10,000 species that have been described to date and have sequenced representative genomes in the public databases in order to be included in our analysis (out of a total 17,000 described species) are largely consistent (>95% of the cases) with the application of the 95% ANI criterion, i.e., genomes showing <95% ANI among themselves have been typically assigned to different species^2^.

## The ANI genetic boundary among microbial species is robust

First, while it is generally accepted that isolation is biased (e.g., favoring copiotrophic vs. oligotrophic or fastidious-to-grow organisms), it is not clear why these biases would affect (or skew) the collection of available public genomes in terms of their ANI values, and would favor closely related genomes (showing >95% ANI) vs. their moderately related close relatives (showing 85–95% ANI), which are -generally speaking- quite similar organisms in terms of biochemistry and physiology. [For instance, *Escherichia coli* and *Salmonella enterica* share about 82–83% ANI and can both grow on the same lab media]. In order to quantify the effects of such biases, if any, one needs to have unbiasedly sampled the diversity that exists in nature and subsequently, assess how well the isolate collection represents this diversity. To the best of our knowledge, such a dataset is not currently available. Instead, the analysis that Murray and colleagues performed in order to prove that isolation biases exist, from a statistical point of view, was to measure the amount of diversity (in terms of branch length in the genome tree) that genomes of a named species represent. They concluded that because 75% of the genomes, on average, represent only about 5% of the intraspecies diversity for several highly sampled species, this result reflects isolation bias. However, a highly homogenous species, with a few more divergent members and a clear ANI gap to its closest relative (distinct) species, would be expected to show the exact same pattern, and it was not made clear how an unbiased (by isolation) species would look like with this respect. Even more relevant for the available genome dataset used (NCBI’s RefSeq), a few divergent relatives that have been misclassified (or misnamed) so they are included in the same species with valid members of the species, or a few chimeric (contaminated) genomes, would account for the results obtained by Murray et al^1^. Our unpublished evaluation as well as the results shown in Fig. 3 in Murray et al^1^. suggest that such misnamed and/or chimeric genomes are frequent enough among the 90,000 genome set to account for the diversity results mentioned above (see also below, for the specific case of *E. coli* as a representative example, and next section for a more detailed rebuttal of this specific point). In short, the random sampling performed by Murray et al., does not prove (or disprove) that biased sampling is responsible for the ANI gap observed. Further, and perhaps more importantly, when one examines the patterns emerging from metagenomic datasets, which is an isolation-free approach, a similar ANI boundary is observed as argued below.

**Fig. 1:**
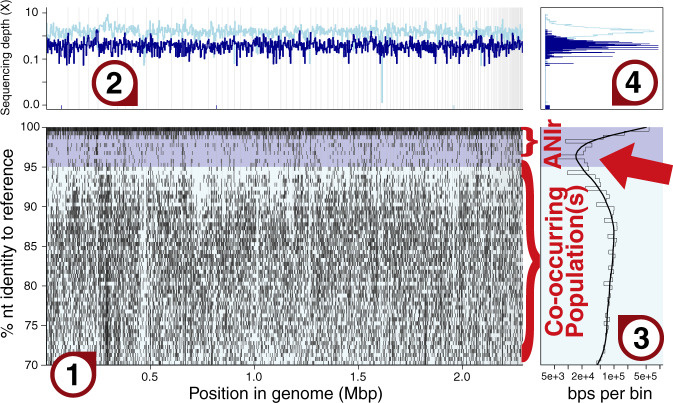
Example of a read recruitment plot. This figure showcases the result of processing a Blastn search of metagenomic short reads (each matching read is represented by a dot in main panel 1) against a reference MAG sequence recovered from the same metagenome (x-axis). **1** Main panel representing the reads recruited (mapped), placed by location (x-axis) and identity (y-axis) across the reference sequence. **2** Sequencing depth (or coverage) across the reference, i.e., how many reads map at each base pair position, in logarithmic scale. Bars lower than the average represent regions with fewer mapped reads, which denote gene content differences. **3** Identity histogram of mapped reads per unit of identity (light gray) and smoothed spline (black), in logarithmic scale. **4** Sequencing depth histogram. Peaks based on the sequencing depth revealed in panel **2** are automatically identified as skewed normal distributions (in red). The background of panels **1** and **3**, and the line colors in panels **2** and **4**, correspond to matches with identity above (dark blue) and below (light blue) a user-defined cutoff. By default, the identity cutoff is set to 95%. Note the area of sequence discontinuity denoted by a decrease, by more than one order of magnitude (x-axis, panel **3**), in the number of reads mapping around 95% identity (red arrow) relative to reads mapping at >98% identity. ANIr is estimated based on reads in the dark blue area only and represents the average nucleotide identity of reads to the reference sequence. The MAG represents an uncultivated member of the *Actinobacteria* phylum that shows about 45% average amino acid (AAI) to *Ilumatobacter coccineus*, its closest related named species with available genome representative(s). The metagenome was obtained from a planktonic sample from 1000 m in the Gulf of Mexico.

In particular, metagenomic studies of natural microbial populations have revealed that bacteria and archaea predominantly form sequence-discrete populations with intrapopulation genomic sequence relatedness typically ranging from ~95 to ~100% ANI depending on the population considered (e.g., younger populations since the last population diversity sweep event show lower levels of intrapopulation diversity). In contrast, ANI values between distinct populations are typically lower than 90%, and genotypes of intermediate genetic relatedness, e.g., showing 85–95% ANI values, are infrequently encountered or coexist in the same samples [Fig. 1 and reviewed in ref. ^5^]. Such sequence-discrete populations were recovered from many different habitats, including marine, freshwater, soils, sediment, human gut, and biofilms, and were typically persistent over time and space [e.g., refs. ^6–10^,] indicating that they are not ephemeral but long-lived entities. The sequence-discrete populations typically harbor substantial intrapopulation gene content diversity as well (i.e., they are rarely clonal), although it has been challenging to robustly assess the extent of this gene diversity based on short read metagenomes^6,9^. Therefore, these populations appear to be “species-like” and may constitute important units of microbial communities^5^.

One way that the sequence-discrete populations have been elucidated is with read-recruitment plots (e.g., Fig. 1). In these plots, the reads of a metagenome (or a metatranscriptome) are mapped against a reference genome sequence that is representative of the population (e.g., an isolate genome or a metagenome-assembled genome, i.e., a MAG), and the mapping patterns reveal the region of sequence-discontinuity (if the latter exists) as well as the level of intrapopulation sequence and gene content diversity^6,11^. Thus, read-recruitment plots provide a thorough and quantitative view of the natural population in a sample and its diversity, and “let the data tell” what the prevailing patterns are, with no biases and/or subjective human interference. Specifically, in the predominant case in which a sequence-discontinuity is observed, reads representing the reference population map evenly (or in an unbiased fashion) across the genome with identities typically (but not necessarily always; see also below) higher than 95% nucleotide identity, whereas sequences representing co-occurring but distinct populations (species) typically show nucleotide identity <90%; reads in the 85–95% nucleotide identity range are generally sparse (e.g., Fig. 1). Recently, our team has advanced the read-recruitment plot tool to provide additional information based on read mapping, such as what is the average coverage of the genome by reads (a proxy for relative metagenome abundance), whether or not co-occurring populations exist in the dataset (sample), and what is their ANI to the reference genome or population as shown in Fig. 1 and further documented in the enveomics collection^12^.

**Fig. 2:**
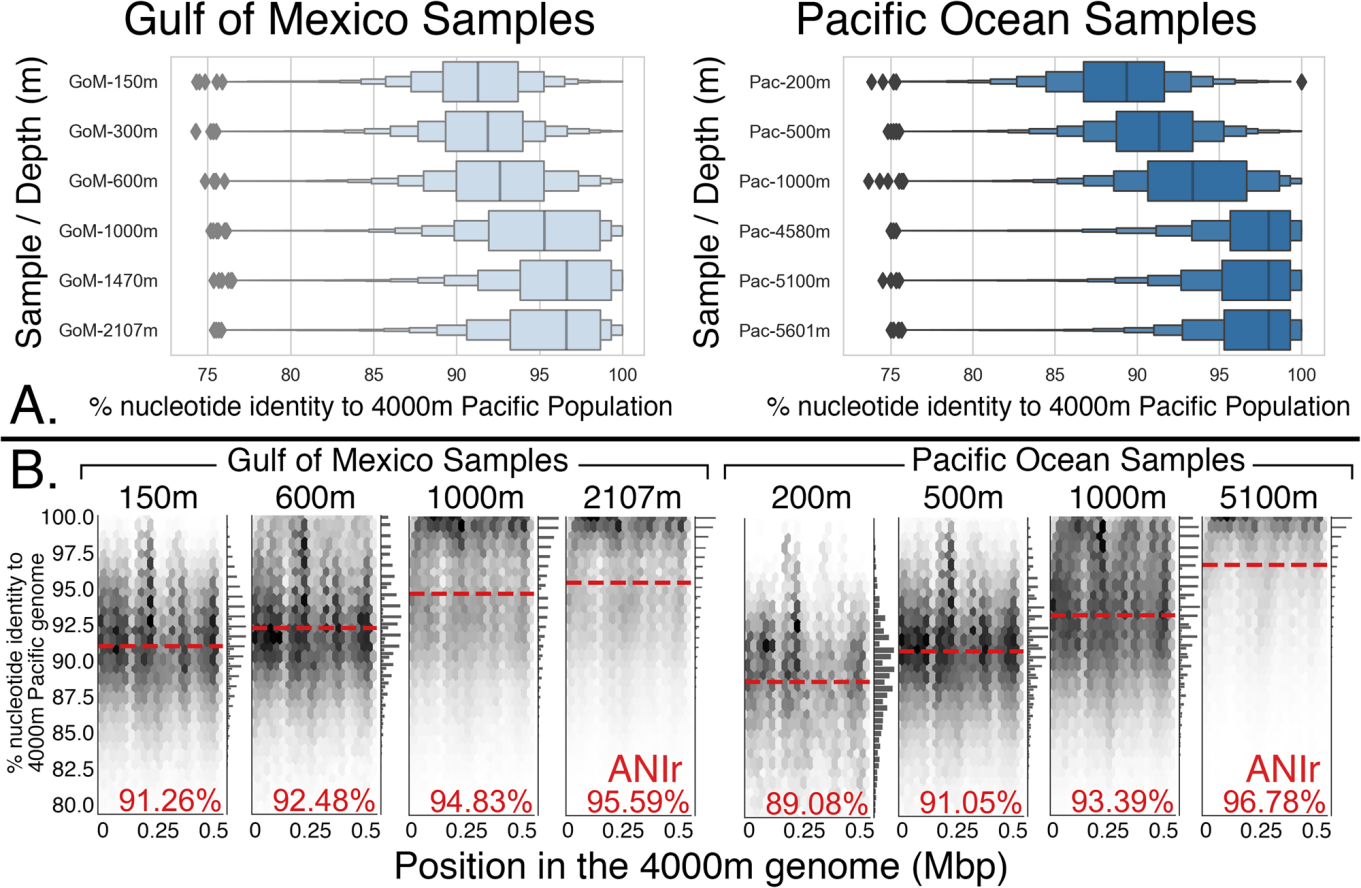
Biogeography of sequence-discrete *Thaumarchaeota* Group I populations. A reference genome representing the thaumarchaeotal population at 4000-m depth in the Pacific Ocean was queried against the previously described metagenomes from six different depths of the Pacific Ocean^29^ and the Gulf of Mexico (our unpublished data). **A** Range in nucleotide identities between the metagenomic read sequences and the genome, represented as letter-value plots^30^, and their vertical line the median (x axis), plotted against the depth that the metagenomic sequences were recovered from (y axis). **B** Read recruitment representation for selected comparisons performed [the uppermost box-plot in the panel **A** represents the distribution of sequence identity values of the reads against the reference genome shown in the leftmost plot in the panel **B**. The plots in panel B are similar to the low, left panel of Fig. 1 but the data points (representing mapped reads) have been binned into a positional, hexagonal heatmap for demonstration purposes. Note that *Thaumarchaeota* are genetically distinct between different depths of the same water column (**A**) but genetically more similar across similar depths in geographically distant locations (**B**), and that if representative genomes or whole-populations from all depths are compared, they will show a range of ANI values between 89 and ~100% among themselves. Note also that the use of short Illumina reads tends to overestimate nucleotide identity (and thus ANIr values) compared to longer Sanger reads or whole/partial genomes used in our previous publications, especially for moderately identical sequences (e.g., in the range of 80–95% nucleotide identity), mostly due to inability of current read mapping algorithms to align such short sequences. Further, the mapping of metagenomic reads to the reference genome was performed with MegaBLAST, in contrast to Blastn in Fig. 1, and MegaBLAST is even less sensitive (but much faster) in finding reads of intermediate identity (e.g., in the range of 70–90% nucleotide identity) compared to Blastn^31^. Therefore, the ANIr values shown are higher than our previous estimates for similar samples (or even Blastn-derived estimates based on the same metagenomic reads) due to this technical limitation, but the diversity patterns across depths remain similar.

Murray and colleagues disregarded all these metagenomic findings and tools by claiming that the genomes recovered from metagenomic datasets by genome binning tools (e.g., MAGs) are biased toward the abundant organisms, and low-abundance genome variants are rarely represented among the recovered genomes. This is partly true^13^, but it fails to consider that abundance is not ecologically neutral (e.g., organisms that show substantially different abundances under the same conditions and are copresent in the same sample are presumably ecologically differentiated from one another) nor is it a technical artifact. Consequently, the question surrounding the 95% ANI gap is not about the nonexistence of intermediate relatives, but rather about their relative rarity in nature. Moreover, this bias does not apply to the individual metagenomic reads. The reads will randomly sample both abundant and rare taxa, e.g., if genomes of intermediate genetic relatedness to the reference genome were collectively abundant, this would have been revealed by the read recruitment plots (by high frequency of reads showing 85–95% nucleotide identity to the reference). However, in the great majority of read recruitment plots performed by us or others (e.g., refs. ^7,14^.) using isolate genomes of abundant or rare taxa, MAGs, or single-cell amplified genomes (SAGs) as reference genomes, the genetic discontinuity (i.e., lack of reads in the 85–95% nucleotide identity range) was evident based on the mapping patterns of reads to these reference genomes. In other words, although reads representing organisms of intermediate identity were often (but not necessarily always) present in the metagenomic dataset, these reads were of much lower frequency than reads of high (>95%) or low (<85%) nucleotide identity to the reference [note that intermediate identity reads/organisms are expected to be completely absent in cases where strong population diversity sweeps/extinctions such as the one represented in Fig. 2B, rightmost plot, have taken place]. Therefore, the picture emerging from isolation-independent, metagenomic datasets is strikingly similar with that based on isolates obtained by Jain and colleagues.

Murray and colleagues also cite two papers^5,15^ led by one of us (Konstantinidis) that reported sequence-indiscrete populations based on metagenomic datasets but did not recognize that these examples were presented by the original studies as the exceptions that confirmed the rule rather than the prevailing pattern of diversity and were attributable to specific (predictable) physicochemical processes that mix distinct populations from different samples and/or habitats (e.g., seawater mixing; see below). Allow us to use one of these examples to further illustrate several key points, including the frequency of intermediate relatedness genotypes. Populations of deep-sea Group I *Crenarchaea* [recently renamed *Thaumarchaeota*^16^] are distinct between different depths of the oceans (Fig. 2B). However, in cases of vertical water mixing such as that observed during ocean upwelling, the populations get mixed, and thus indiscrete (continuous) “populations” may appear temporarily in samples from such mixed waters. When waters get stratified, as they do in summer, the deep-sea populations are absent from surface waters and vice versa^17,18^. Thus, depth-stratified, sequence-discrete populations appear to be the predominant pattern throughout the ocean’s water column as well.

Furthermore, if one samples just one genome representative from each thaumarchaeotal population (or distinct depth biome) and does the ANI comparison among the resulting subsample of genomes, which is analogous to the subsampling that Murray and colleagues did on the isolate genomes to show the lack of the ANI boundary, then a genetic continuum may be revealed [Fig. 2A and in ref. ^17^]. However, this is obviously a biased sampling because the local abundance information, which is linked to the ecophysiology of the organisms, is not considered; e.g., in the deep-sea samples, surface populations are rarely found and, if they are ever found, they are temporarily associated with sinking particles and/or water mixing events. It also follows that the deep and surface populations should not be considered the same species, even if they perform similar metabolic functions in terms of energy generation, because under the same conditions (e.g., stratified/stable waters) only one of them dominates/thrives at its preferred depth^17^. That is, the populations are apparently not interchangeable since under the same conditions, they do not coexist but, instead, one has much lower abundance (just barely surviving), if detected at all, than the other one. In summary, genetically-related yet ecologically and genetically distinct populations (e.g., preference for different depths for marine *Thaumarchaeota*), should not be considered the same species, a point not considered by Murray and colleagues. Similar concepts and patterns most likely apply to other taxa and habitats, such as to *E. coli* and its closely-related environmental genomes/clades^19^ cited by Murray and colleagues. These environmental *E. coli*-like clades have never been found to be abundant in the gut (or feces) of human or warm-blooded animals to the best of our knowledge (a couple of the clades are thought to be abundant in birds), contrasting with the typical *E. coli* commensal genomes, and are genetically distinct (e.g., their ANI values to typical *E. coli* are 90–93% vs. >95% among typical *E. coli* genomes). Hence, these clades are arguably ecologically and genetically differentiated enough to represent different species than typical *E. coli*, albeit no systematic effort to taxonomically describe them as novel (distinct) species has yet taken place. Lacking such taxonomic description, Murray and colleagues considered these environmental genomes to be members of the *E. coli* species. However, even based on the commonly used genomic standards to define (named) species (e.g., 95% ANI threshold^4^), without considering ecological differentiation, each of the environmental clades would represent a distinct species of the *Escherichia* genus. Notably, including these environmental genomes as *E. coli* species would account, at least partly, for the main finding of Murray et al^1^., that a few genomes of the species represent the great majority of the intraspecies diversity (in terms of branch lengths; related to the point above) and obviously, does not prove the existence of isolation biases (but rather the effect of how organisms are named).

As is also obvious from the abovementioned examples, the existence of intermediate relatedness/ANI genotypes was noted earlier by Konstantinidis and colleagues in both the free-living marine *Thaumarchaeota*^6^ as well as other taxa, including sponge-associated symbiotic organisms^15^ and the *E. coli* group^19^. Murray and colleagues basically confirmed these earlier observations based on different genomic data. Indeed, these findings are also probably consistent with what should have been expected for prokaryotic genomes based on their (known) great genomic adaptability (e.g., hyper-mutator phenotypes and high survival rates based on low metabolic activity) and fluidity (e.g., extensive horizontal gene transfer and gene loss), and their immense sequence space. However, without the relative abundance and ecological preference information, it is arbitrary to group genomes into the same or different species^4^ and thus, discuss the existence (or not) of boundaries among species. When these factors are considered together with genetic relatedness, microbial species appear to predominantly exist and be genetically (and presumably ecologically) distinct from each other^5^. To provide an analogous example from higher eukaryotic organisms, consider the hypothetical case that a researcher samples just one individual human and one chimpanzee from the whole Earth and examines their genome sequences (which is similar to what Murray and colleagues did for several prokaryotic species such as the *E. coli* and its closely-related environmental clades). Based on their high genetic relatedness, one could (misleadingly) conclude that the two organisms belong to the same species and show a range of intraspecies ANI values between 100% (self-match) to ~98.5% (the ANI between human and chimpanzee genomes). However, with more sampling of individuals and assessment of their (different) ecophysiology, it would become clear that these individuals belong to two distinct species (*Homo sapiens* and *Pan troglodytes*) by most metrics, including higher intra- vs. inter-species ANI values (and intermediate genotypes are rarely found, if ever).

It is also important to clarify one additional issue. Jain and colleagues, and our previous papers [e.g., ref. ^20^,] cited by Murray and colleagues, didn’t claim that the 95% ANI threshold is a hard cut-off or that it is a “first principles” value. On the contrary, the claim is that we observe this sequence boundary in nature, likely as an emerging property of many eco-evolutionary forces at play, and that this threshold may slightly differ for different taxa. This is similar to how the 70% DNA-DNA hybridization standard, which has guided many species-level taxonomic descriptions in the last four decades, was proposed and should have been used^4^. For instance, some populations affiliated with marine *Prochlorococcus marinus* (photosynthetic Cyanobacteria)^6^, “*Ca*. Pelagibacter ubique” (SAR-11; oligotrophic Proteobacteria)^21^, and the *Thaumarchaeota* mentioned above [Fig. 2 and in ref. ^17^] show large intrapopulation sequence diversity, probably due to unique ecological niche(s) they have occupied for long evolutionary times compared to other marine taxa (i.e., they lack direct competition), and thus this threshold is around 90–92% ANI for these populations. In contrast, several more recently emerged pathogens like *Bacillus anthracis*^22^ show limited intrapopulation/species diversity (ANI values >99%). Hence, the area of genetic discontinuity may vary, depending on the taxa considered and their unique ecophysiologic and evolutionary characteristics, and 95% ANI appears to be the genetic level that distinguishes most natural discrete populations^6^ and named species^2^, but not necessarily all. For these reasons, taxon descriptions should not be based on a single metric or threshold but the careful investigation of ecological and functional data together with genetic relatedness (e.g., ANI values).

In summary, with increasingly more genomic and metagenomic data accumulating in the public databases at the time of this writing, we will see in the near future if the recommended ANI standard will have to be adjusted and in which direction. Murray and colleagues did not disprove the existence of a genetic boundary between species with their analysis; rather, their results simply raised the possibility of alternative explanations. As it was argued above, however, these explanations already exist in the literature and hopefully, contributions such as the one by Murray and colleagues will stimulate further research on the topic in the future. In our view, the important question to study in order to truly advance the species concept and further corroborate the existence (or lack) of genetic and/or ecological boundaries among species is how the intermediate identity genotypes—when present—differentiate functionally and ecologically from their close relatives. The relative importance of genetic (e.g., recombination) and ecological factors (e.g., niche overlap and functional differentiation) in maintaining (or not) sequence-discrete populations needs to be quantified as well. To date, a few taxa have been studied in this respect, but the picture is currently far from complete to allow for robust, universally applicable conclusions to emerge.

## Rebuttal of minor points raised in the Murray et al Matters Arising

Murray and colleagues, in backing up their argument about lack of a genetic boundary, have not considered several key concepts as noted below.

1. (from Murray et al^1^) “There is much evidence against the existence of a universal genetic boundary for microbial species. First, the molecular substitution rate is highly variable across species.”
  This is only superficially true, since the variation is large only in response to specific molecular mechanisms (that are not unlimited in number or effect), while fixation rates (not substitutions) depend on effective population sizes and other population-specific characteristics. Therefore, one would expect some variation, but mostly tightly centered around a mean and maybe with a large tail. Accordingly, general metrics such as 16S rRNA gene phylogenetic distance and ANI apply well across prokaryotes, and we should expect to find universal properties such as a genetic boundary.
2. (from Murray et al^1^) “Secondly, selection and recombination are thought to be the main cohesive forces driving the formation of genetic clusters. Although recombination rate can be influenced by sequence similarity, there is no correlation between the recombination rate and ANI in bacteria (12)”.
  That is almost exactly opposite to what the cited Bobay and Ochman paper says. That paper discusses ANI only very briefly, and uses a nonstandard definition of ANI, so it is not directly applicable. However, the paper does say: “The most divergent members in the biological species that we recognized usually average no more than 5% difference in the nucleotide sequences of their core genes; however, there is a long tail to the distribution of DNA identity values”. Since the “biological species that they recognized” are based on recombination rates, this means that there is a correlation and it actually tends to align with the 95% ANI (5% divergence), although Bobay and Ochman also recognize some extreme outliers (long tail). Moreover, Olm and colleagues actually do test this directly against ANI, and Murray and colleagues did not discuss those results in their paper^10^.
3. Murray and colleagues attributed the bimodal distribution in ANI values observed by Jain and colleagues to the high frequency of redundant genomes in the public databases. While their definition of “redundant genome” was not provided, it is important to note that most genomes (about 75% of the total) showing >95% ANI (same species assignment) show <99% ANI with each other (e.g., Fig. 3C in Jain et al^2^. 2018) and have substantial gene content differences. Therefore, these genomes do not represent the same strain or clone from a few pathogen outbreaks but substantially divergent, distinct strains of the same species [Note also that 1% difference in ANI corresponds to a long evolutionary time since the last common ancestor, e.g., >10,000 years for *E. coli*^23^]. Presumably, most of these strains were obtained by different labs, studies and/or samples, and thus represent reliable and robust sampling of the intraspecies diversity.
4. Murray and colleagues suggested that the experiment that Jain and colleagues performed in order to account for the overrepresentation of a few species in the 90 K genome set, i.e., subsample five genomes at random from all 750 species that had five or more genomes available, cannot correct for biased sampling if the original (complete) dataset is biased. This experiment showed a similar bimodal distribution in ANI in values among the sampled genomes (Supplementary Fig. 10 in Jain et al^2^. 2018), albeit with a smaller right peak (ANI values >95%), as expected due to the subsampling. It can be argued that this subsampling performed by Jain and colleagues is less biased than sampling only two, most divergent genomes, from each species (or just one genome per species) performed by Murray and colleagues for assessing whether or not an ANI boundary exists (for other research questions, the subsampling performed by Murray and colleague could be appropriate). Finally, Jain et al^2^., performed an additional relevant experiment that is also important to mention. In particular, they removed the top-n largest 95%-ANI clusters and monitored the effect on the frequency of ANI values that made up the three key bins: <83%, 83–95%, ≥95% (Suppl. Fig. 9). The result was that the rightmost peak started decreasing in magnitude with more clusters removed (as expected, and also as observed by Murray and colleagues) but, importantly, the 83–95% region did not change much and, in fact, decreased in magnitude after about ten clusters were removed. If the boundary was simply an artifact of the most highly sequenced species, the fraction of pairs in the 83–95% region should monotonically increase with cluster removal, which is the opposite of what Jain and colleagues observed.
5. Murray and colleagues offer Fig. 2 of their manuscript as the visual demonstration that genome pairs of intermediate ANI values (e.g., 85–95%) are often present within the most studied named species. While this is admittedly true, as the discussion above on intermediate identity genotypes based on metagenomic datasets also suggests, Fig. 2 does show that for most—if not all—of these species the genome pairs with high ANI values (e.g., >95%) are comparatively much more prevalent than the intermediate ANI pairs, which is consistent with our previous study^2^. Further, for several species shown such as members of the *Streptococcus* genus, intermediate genotypes are completely absent. In addition, there are apparently technical issues with this figure (e.g., misnamed/misclassified and chimeric genomes) since—as an example—the *E. coli* row should have had many more pairs to the right of the distribution (i.e., more red color in the >95% ANI range) given that the available intermediate “environmental” *E. coli*-like genomes are only a few (fewer than a dozen or so) compared to the thousands of typical *E. coli* genomes [unless, *Salmonella* spp. genomes were also included in the *E. coli* data, which would not be consistent with the legend of the figure since *Salmonella* spp. are clearly distinct named species; further, inclusion of *Salmonella* genomes would artificially flatten the datapoint distribution].
6. Finally, Murray and colleagues provided the diversity of *E. coli* isolate genomes recovered by 16S rRNA gene sequences relative to that of all *E. coli* 16S rRNA gene sequences found in the GreenGenes database (Supplementary Fig. 2) as proof of biased representation of the total natural diversity by genome sequences. However, the sequenced genomes cannot capture the total diversity because they represent a subset of the total available isolates. More importantly, it is not clear if the additional diversity is based on isolates (hence, no isolation bias) or environmental surveys; and, if the latter, many (most?) of the available sequences are likely single-pass sequencing reactions (not the resulting consensus of overlapping sequences) that are prone to sequencing errors and artifacts. In other words, Fig. S2 cannot be fully evaluated without an assessment of sequencing errors in the GreenGenes sequences, which are likely not rare, especially for environmental sequences. It is also important to point out that *E. coli* has seven rRNA operons, six of which are nearly identical to each other (0–2 single nucleotide differences) and one more divergent operon (~99% nucleotide identity to the rest)^24^. Such rRNA operon structure is known to be challenging for PCR amplification and sequencing, often producing chimeric or erroneous sequences, especially during single pass sequencing reactions.

## Methods

All Illumina sequenced metagenomic datasets were processed with the same pipeline to ensure consistent results. Raw reads from each dataset in fastq format were processed using Trimmomatic version 0.39^25^ with settings ILLUMINACLIP AllAdapters-PE.fa:2:30:10:2:keepBothReads LEADING:3 TRAILING:3 MINLEN:36 to remove sequencing adapters and low quality base calls. Fastq files were converted to fasta format using the FastQ.toFastA.awk script from the enveomics collection^12^. Read mapping to reference genome sequences was performed using Blast+ version 2.10.1^26^ and either “blastn” or “megablast” for the “-task” flag and additional settings “-evalue 0.01 -max_target_seqs 10 -perc_identity 70 -outfmt ‘6 qseqid sseqid pident length mismatch gapopen qstart qend sstart send evalue bitscore qlen slen’.” Tabular Blast results were filtered for a minimum read length of 70 base pairs and an alignment length/read length greater than or equal to 0.9 using a custom Python script (https://github.com/rotheconrad/GoM). The recruitment plot shown in Fig. 1 was generated using filtered tabular blast output and the BlastTab.catsbj.pl and BlastTab.recplot2.R scripts from the enveomics collection^12^ with additional labels added using Adobe Illustrator. The plots shown in Fig. 2 were generated using data from column 3 (pident) from the filtered tabular blast output and a custom Python script (https://github.com/rotheconrad/GoM) and the Matplotlib version 3.3.2^27^ and Seaborn version 0.11.0^28^ packages.

### Reporting summary

Further information on research design is available in the Nature Research Reporting Summary linked to this article.

## Supplementary information

Reporting Summary

## Data Availability

The six Pacific Ocean metagenomes used in Fig. 2 are available in NCBI, under accession numbers SRR5002329, SRR5002314, SRR5002320, SRR5788244, SRR5788420, and SRR5788153. The Group I thaumarchaeotal genome sequence used in Fig. 2 is publicly available as part of the Konstantinidis and DeLong, ISME J. 2008 article. This genome sequence as well as the genome sequence used as reference in the read recruitment plot of Fig. 1 and the Gulf of Mexico metagenomes used in Figs. 1 and 2 are also available through http://enve-omics.ce.gatech.edu/data/gom_depth.

## Code availability

The read recruitment plot presented in Fig. 1 was produced using the corresponding scripts of the enveomics scripts collection^12^.
