# Supplementary material for: Reply to: “Re-evaluating the evidence for a universal genetic boundary among microbial species”
Source: Nat Commun. 2021 Jul 7;12:4060. doi: 10.1038/s41467-021-24129-1 (PMC8263725; doi:10.1038/s41467-021-24129-1)
Supplement: Supplementary file 1 — Reporting Summary [file 41467_2021_24129_MOESM1_ESM.pdf]

nature research

Corresponding author(s): NCOMMS-20-42232B
Corr. Author: Konstantinos T. Konstantinidis

Last updated by author(s): 2021/05/18

Reporting Summary

Nature Research wishes to improve the reproducibility of the work that we publish. This form provides structure for consistency and transparency in reporting. For further information on Nature Research policies, see our Editorial Policies and the Editorial Policy Checklist.

Statistics

For all statistical analyses, confirm that the following items are present in the figure legend, table legend, main text, or Methods section.

n/a	Confirmed	
☐	☒	The exact sample size (*n*) for each experimental group/condition, given as a discrete number and unit of measurement
☐	☒	A statement on whether measurements were taken from distinct samples or whether the same sample was measured repeatedly
☒	☐	The statistical test(s) used AND whether they are one- or two-sided<br>*Only common tests should be described solely by name; describe more complex techniques in the Methods section.*
☒	☐	A description of all covariates tested
☒	☐	A description of any assumptions or corrections, such as tests of normality and adjustment for multiple comparisons
☐	☒	A full description of the statistical parameters including central tendency (e.g. means) or other basic estimates (e.g. regression coefficient) AND variation (e.g. standard deviation) or associated estimates of uncertainty (e.g. confidence intervals)
☒	☐	For null hypothesis testing, the test statistic (e.g. *F*, *t*, *r*) with confidence intervals, effect sizes, degrees of freedom and *P* value noted<br>*Give P values as exact values whenever suitable.*
☒	☐	For Bayesian analysis, information on the choice of priors and Markov chain Monte Carlo settings
☒	☐	For hierarchical and complex designs, identification of the appropriate level for tests and full reporting of outcomes
☒	☐	Estimates of effect sizes (e.g. Cohen's *d*, Pearson's *r*), indicating how they were calculated

Our web collection on statistics for biologists contains articles on many of the points above.

Software and code

Policy information about availability of computer code

Data collection	The pieces of software used were the read recruitment plots of the enve-omics script collection and the ani calculator. All pieces of software are freely available on our website (http://enve-omics.ce.gatech.edu) for online analysis or download, and clearly mentioned in the manuscript and with the appropriate references cited.
Data analysis	All data used in our manuscript is publicly available and open access on our website (http://enve-omics.ce.gatech.edu) and NCBI. Also this information is clearly mentioned in a "Data release" sections of the manuscript. The recruitment plot shown in Figure 1 was generated using filtered tabular blast output and the BlastTab.catsbj.pl and BlastTab.recplot2.R scripts from the enveomics collection with additional labels added using Adobe Illustrator. The plots shown in Figure 2 were generated using data from column 3 (pident) from the filtered tabular blast output and a custom Python script (https://github.com/rotheconrad/GoM) and the Matplotlib version 3.3.2 27 and Seaborn version 0.11.0 28 packages (also cited appropriately).

For manuscripts utilizing custom algorithms or software that are central to the research but not yet described in published literature, software must be made available to editors and reviewers. We strongly encourage code deposition in a community repository (e.g. GitHub). See the Nature Research guidelines for submitting code & software for further information.

Data

Policy information about availability of data

All manuscripts must include a data availability statement. This statement should provide the following information, where applicable:

- Accession codes, unique identifiers, or web links for publicly available datasets
- A list of figures that have associated raw data
- A description of any restrictions on data availability

The six Pacific Ocean metagenomes used in Figure 2 are available in NCBI, under accession numbers SRR5002329, SRR5002314, SRR5002320, SRR5788244,

SRR5788420, and SRR5788153. The Group I thaumarchaeotal genome sequence used in Figure 2 is publicly available as part of the Konstantinidis and DeLong, ISME J. 2008 article. This genome sequence as well as the genome sequence used as reference in the read recruitment plot of Figure 1 and the Gulf of Mexico metagenomes used in Figures 1 and 2 are also available through http://enve-omics.ce.gatech.edu/data/gom_depth.

Field-specific reporting

Please select the one below that is the best fit for your research. If you are not sure, read the appropriate sections before making your selection.

☐ Life sciences ☐ Behavioural & social sciences ☒ Ecological, evolutionary & environmental sciences

For a reference copy of the document with all sections, see nature.com/documents/nr-reporting-summary-flat.pdf

Ecological, evolutionary & environmental sciences study design

All studies must disclose on these points even when the disclosure is negative.

Study description	Our study used previously published data and there is no new experimental data reported as part of the study. We report only re-analysis of previously published data.
Research sample	Available microbial metagenomes published by previous studies.
Sampling strategy	Not applicable
Data collection	Not applicable
Timing and spatial scale	Not applicable
Data exclusions	Not applicable
Reproducibility	Methods are described in detail in the Methods section and should be reproducible by anybody with minimum/beginning knowledge of bioinformatics.
Randomization	Not applicable
Blinding	Not applicable

Did the study involve field work? ☐ Yes ☒ No

Reporting for specific materials, systems and methods

We require information from authors about some types of materials, experimental systems and methods used in many studies. Here, indicate whether each material, system or method listed is relevant to your study. If you are not sure if a list item applies to your research, read the appropriate section before selecting a response.

Materials & experimental systems

n/a	Involved in the study	
☒	☐	Antibodies
☒	☐	Eukaryotic cell lines
☒	☐	Palaeontology and archaeology
☒	☐	Animals and other organisms
☒	☐	Human research participants
☒	☐	Clinical data
☒	☐	Dual use research of concern

Methods

n/a	Involved in the study	
☒	☐	ChIP-seq
☒	☐	Flow cytometry
☒	☐	MRI-based neuroimaging
